# Body composition associations with muscle strength in older adults living in Auckland, New Zealand

**DOI:** 10.1371/journal.pone.0250439

**Published:** 2021-05-28

**Authors:** Anne N. Hiol, Pamela R. von Hurst, Cathryn A. Conlon, Owen Mugridge, Kathryn L. Beck

**Affiliations:** School of Sport, Exercise and Nutrition, Massey University, North Shore City, New Zealand; Clinca Geriatrica, ITALY

## Abstract

**Background:**

Aging is associated with decreases in muscle strength and simultaneous changes in body composition, including decreases in muscle mass, muscle quality and increases in adiposity.

**Methods:**

Adults (n = 369; 236 females) aged 65–74 years living independently were recruited from the cross-sectional Researching Eating Activity and Cognitive Health (REACH) study. Body fat percentage and appendicular skeletal muscle mass (ASM) (sum of lean mass in the arms and legs) were assessed using Dual-energy X-ray Absorptiometry (Hologic, QDR Discovery A). The ASM index was calculated by ASM (kilograms) divided by height (meters) squared. Isometric grip strength was measured using a hand grip strength dynamometer (JAMAR HAND).

**Results:**

Linear regression analyses revealed that muscle strength was positively associated with the ASM index (R^2^ = 0.431, p < 0.001). When exploring associations between muscle strength and muscle mass according to obesity classifications (obesity ≥30% males; ≥40% females), muscle mass was a significant predictor of muscle strength in non-obese participants. However, in participants with obesity, muscle mass was no longer a significant predictor of muscle strength.

**Conclusions:**

Body fat percentage should be considered when measuring associations between muscle mass and muscle strength in older adults.

## 1. Introduction

Globally and in New Zealand the proportion of older adults is increasing [1]. In 2006, 12.3% of New Zealand population was older than 65 years; this percentage is expected to increase to 19.9% by the year 2026 and to more than 26.3% by 2051 [2].

In the aging New Zealand population, falls are both the most common and costliest cause of injury [3]. In 2016 the rate of one or more Accident Compensation Corporation (ACC) claims for a fall-related injury was 216 per 1,000 in people aged 50 and over. People aged 85 and over were twice as likely as 50–64-year olds to have an ACC claim for a fall-related injury [4].

Evidence pooled from a large number of studies has shown that one of the most common risk factors for falls is low muscle strength [5–7]. Muscle strength refers to the amount of force a body can produce to perform normal daily household, work related, and recreational activities. Muscle strength increases with age and then significantly decreases after 40 and 50 years old for women and men, respectively [8].

Aging is also associated with changes in body composition, including decreases in muscle mass, muscle quality and increases in adiposity [9, 10]. These changes occur simultaneously with a decline in muscle strength.

Cross-sectional studies have demonstrated that muscle mass is correlated with muscle strength [11–13]. However, longitudinal studies have shown that changes in muscle mass explain only a small portion (~5%) of the changes in strength in older adults [10, 14]. This indicates that other factors may preserve muscle strength during aging, one of which may be age related changes in adiposity.

The prevalence of obesity in older adults living in New Zealand has increased [15, 16]. Body Mass Index (BMI) is commonly used to assess obesity as it is easily measured and does not require costly equipment. One cross-sectional study found that muscle strength was positively associated with BMI in underweight, normal, overweight and obese older men and women [17]. In contrast, another study found low muscle strength had a negative association with BMI (overweight/obese) in older adults [18]. BMI has poor diagnostic accuracy for identifying older adults with obesity [19, 20], which might account for the contrary findings. Body fat percentage (%BF) is a more accurate reflection of obesity in older adults. Several studies have demonstrated that higher body fat percentage is associated with lower muscle strength [10, 21] and lower muscle mass in older adults [22, 23]. This finding suggests that body fat percentage may contribute to the relationship between muscle strength and muscle mass.

Body composition is related to muscle strength, however there is very limited evidence regarding the contribution of obesity classification based on body fat percentage when investigating the relationship between muscle strength and muscle mass in older adults. The aim of this research was to examine the relationship between muscle strength, muscle mass, and body fat percentage in older adults living in Auckland, NZ.

## 2. Materials and methods

### 2.1. Study design

This study was a secondary aim of the Researching Eating, Activity and Cognitive Health (REACH) Study. The main objective of the REACH Study was to investigate dietary patterns and associations with cognitive function and metabolic syndrome in older adults. Ethical approval was granted by the Massey University Human Ethics Committee: Southern A, Application 17/69 and all participants provided written informed consent. Further information regarding the REACH study protocol can be found elsewhere [24]. The study took place in the Human Nutrition Research Unit at Massey University’s Auckland Campus, New Zealand (NZ).

### 2.2. Study participants and procedures

Participants included men and women aged 65–74 years, living independently in Auckland, NZ. Exclusion criteria were a diagnosis of dementia or any condition which may impair cognitive function (e.g. traumatic head injury, stroke), medication which may influence cognitive function, colour blindness, or any other event in the last two years which had a substantial impact on dietary intake or cognitive function. Participants who registered their interest in the REACH study were provided with an information sheet and completed an online screening questionnaire to determine their eligibility to take part. If the inclusion criteria were met, participants were invited to participate in the study.

### 2.3. Data collection

All participants visited the Human Nutrition Research Unit on one occasion for collection of data as part of the wider REACH study. Socio-demographic information including age and gender were collected through written questionnaires. Data quality was ensured by checking questionnaires for completeness.

Height and weight measurements were undertaken using standardised techniques adapted from the International Society for the Advancement of Kinanthropometry (ISAK) protocol. Height was measured to the nearest 0.1 cm using a stadiometer (SECA). Weight was measured with the participant in light clothing, using floor scales (Wedderburn). BMI was calculated using body weight in kilograms divided by height (metres) squared.

Body composition values were ascertained from a total body dual-emission X-ray absorptiometry (DXA) scan (Hologic, QDR Discovery A). The machine was checked and calibrated daily in line with the standard operating procedure recommendations. All scanning and analysis procedures were performed by a trained operator. After removal of shoes and jewelry, participants adopted a supine position with arms to the side [25]. Participants were then scanned as per established recommendations [26, 27], with the standard mode scan taking approximately eight minutes to complete. The values for body composition outcomes were determined from the ratio of soft tissue attenuation of two X-ray energy beams for each pixel containing a minimal amount of soft tissue but no significant bone. Body fat percentage was calculated by dividing total fat mass by the sum of bone, lean and fat mass [21]. Regional analyses were performed and appendicular skeletal muscle mass (ASM) was calculated as the sum of mineral-free lean mass of the arms and legs [28, 29]. The ASM index was calculated by ASM (kilogram) divided by height (meters) squared [30]. Low muscle mass was defined as an ASM index <7 kg/m^2^ (men) and <5.5 kg/m^2^ (women) [31].

Isometric grip strength was measured in both hands using an adjustable hand grip strength dynamometer (JAMAR HAND) [32]. The participant was seated on a standard straight back chair without arm rests, and with elbow, hips and knees at 90° angles. All participants were instructed to squeeze the handle as hard as they could upon a verbal signal from the researcher. Verbal encouragement was provided throughout the period of effort which did not exceed 10 seconds. Three measurements were taken for each hand, alternating right/left to permit muscular recovery between replicate trials. Results were recorded in kilograms (kg), the mean of three trials for each hand was recorded and the highest value of the two means was used for further analyses [32, 33]. Low muscle strength was defined as hand grip strength <27 kg in men and <16 kg in women [31].

### 2.4. Statistical analysis

Continuous data were assessed for normality using Shapiro Wilcoxon tests and visual assessment of histograms. Descriptive statistics were reported as means ± SD for parametric data, and frequencies and percentages for categorical data. Differences between groups were analysed using independent t-tests for parametric data, and the chi-square test of independence for categorical data.

The measurements were categorized into two groups according to sex. The measurements were further categorized according to body fat percentage into two groups. Using body fat percentage, obesity categories were defined as ≥30% fat (males) and ≥40% fat (females) [34]. Classification of obesity using BMI was according to the categories suggested by the World Health Organization: underweight (BMI<18.5kg/m^2^), normal weight (18.5–24.9), overweight (25–29.9), and obese (30+) in both men and women [35, 36].

A multiple linear regression analysis was performed to determine body composition parameters predicting muscle strength in males and females. Adjusted r, standard error values, and multicollinearity statistics were used to identify the most appropriate equations. This analysis was undertaken in males and females according to obesity classifications based on body fat percentage.

All statistical analyses were completed using the statistical software IBM SPSS version 26. Results were considered significant at p < 0.05.

## 3. Results

Three hundred and sixty-nine participants (n = 236 females) were included in the analyses. Descriptive statistics for the study population according to sex are presented in Table 1. The mean ± SD age of participants was 69.7 ± 2.6 years. Males were taller, heavier and had a lower body fat percentage than females, but these differences were not significant. The mean BMI for this study population fell in the overweight BMI category (26.34 ± 4.6 kg/m^2^). Using BMI categories, 16.5% and 16.1% males and females were classified as obese. In males and females, 9.8% and 23.7% respectively were classified as obese using body fat percentage categories [34]. The prevalence of low muscle mass was 2.3% and 6.4%, and the prevalence of low muscle strength was 1.5% and 4.7% in males and females respectively. Appendicular skeletal muscle mass index and muscle strength were higher in males compared with females, this difference was significant for muscle strength only (Table 1).

**Table 1:** Characteristics of study participants by sex^a^^,^^b^.

Characteristics		Total	Males	Females	p-value
		n = 369	n = 133	n = 236	
**Age (years)**		69.67 ± 2.57	70.16 ± 2.42	69.39 ± 2.62	0.151
**Height (m)**		1.67 ± 0.09	1.76 ± 0.07	1.63 ± 0.06	0.184
**Weight (kg)**		73.98 ± 15.05	83.09 ± 13.85	68.84 ± 13.18	0.578
**BMI (kg/m**^**2**^**)**		26.34 ± 4.59	26.85 ± 4.01	26.06 ± 4.87	0.066
**BMI categories n (%)**	Underweight	3 (0.8)	1 (0.8)	2 (0.8)	0.019*
	Normal weight	148 (40.1)	40 (30.1)	108 (45.8)	
	Overweight	158 (42.8)	70 (52.6)	88 (37.3)	
	Obese	60 (16.3)	22 (16.5)	38 (16.1)	
**Body fat percentage (%)**		31.84 ± 7.47	24.48 ± 4.41	36.0 ± 5.34	0.067
**Body fat percentage categories n (%)**	Non-obese	299 (81.0)	120 (90.2)	179 (75.8)	< 0.01**
	Obese	69 (18.7)	13 (9.8)	56 (23.7)	
**ASMI (kg/m**^**2**^**)**		7.59 ± 1.38	8.89 ± 7.59	6.85 ± 0.96	0.644
**ASMI categories n (%)**	Normal	351 (95.1)	130 (97.7)	221 (93.6)	< 0.01**
	Low	18 (4.8)	3 (2.3)	15 (6.4)	
**Grip strength (kg)**		30.56 ± 9.78	40.97 ± 7.67	24.69 ± 4.55	< 0.01**
**Grip strength categories n (%)**	Normal	356 (96.5)	131 (98.5)	225 (95.3)	< 0.01**
	Low	13 (3.5)	2 (1.5)	11 (4.7)	

^a^Continuous values are expressed as mean ± standard deviation.

^b^Categorical values are expressed as frequency (percentage). Sex difference at **p < 0.01, *p < 0.05. BMI = body mass index. ASMI = appendicular skeletal muscle mass index. Classification of obesity using BMI was according to categories suggested by the World Health Organization: underweight (BMI<18.5kg/m^2^), normal weight (18.5–24.9), overweight (25–29.9), and obese (30+) in both men and women. Using body fat percentage, obesity categories were defined as ≥30% fat (males) and ≥40% fat (females). Low muscle mass was defined as an ASM index <7 kg/m^2^ (men) and <5.5 kg/m^2^ (women). Low muscle strength was defined as handgrip strength <27 kg in men and <16 kg in women.

**Fig 1:**
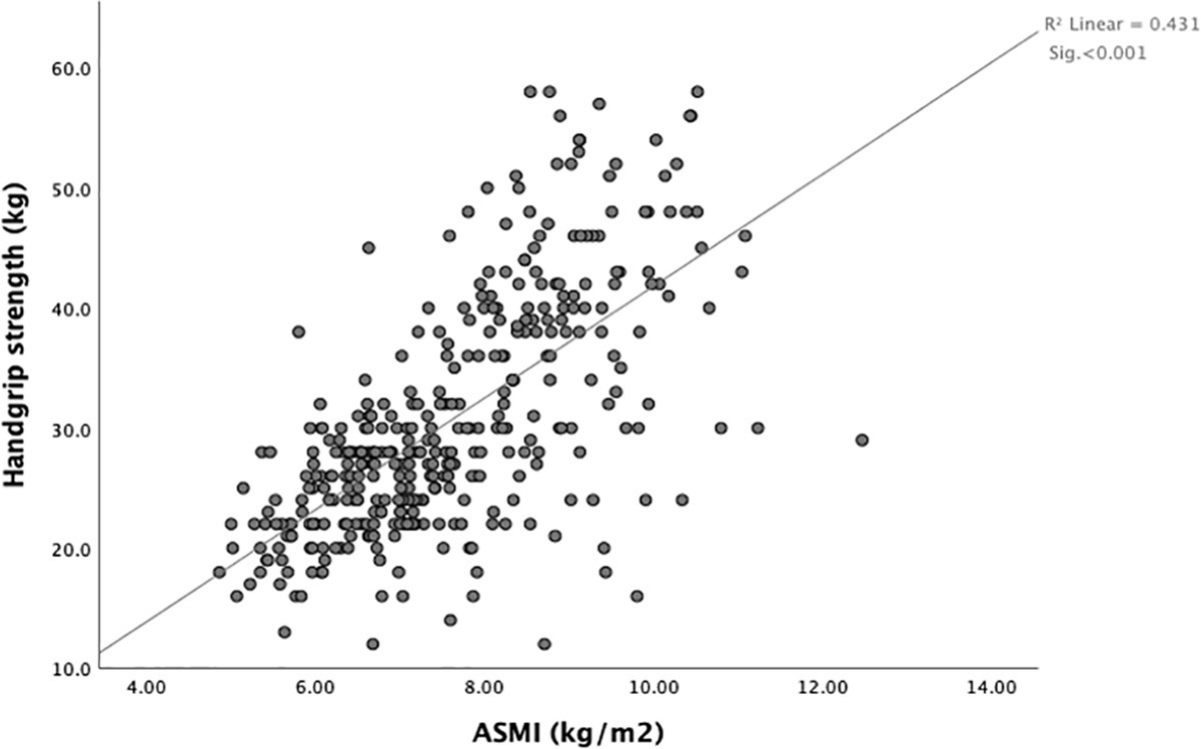
Association between muscle strength and mass in females and males (n = 369).

In both males and females, muscle strength was positively associated with appendicular skeletal muscle mass index (R^2^ = 0.431, p < .001) (Fig 1).

When stratified by sex, the association was still significant, but lower (females R^2^ = 0.040, p = 0.002; males R^2^ = 0.055, p = 0.006) (Tables 2 and 3), with the addition of %BF increasing the ability of the model to predict muscle strength slightly (females R^2^ = 0.116, p < 0.001; males R^2^ = 0.097, p < 0.001).

**Table 2:** Results of multiple linear regression modelling on the relationship between muscle strength, mass and body fat percentage in older females.

Model	Coefficient (B)	Standard error B	95% CI	Standardised β	R^2^	P-Value
**Model 1**					0.04**	0.002
**Constant**	18.217	2.107	14.066, 22.368			
**ASMI**	0.945	0.304	0.345, 1.545	0.199		
**Model 2**					0.116**	< 0.001
**Constant**	24.613	2.480	19.727, 29.498			
**ASMI**	1.288	0.303	0.692, 1.885	0.271		
**%BF**	- 0.243	0.054	- 0.350, - 0.136	- 0.285		

ASMI = appendicular skeletal muscle mass index. %BF = body fat percentage. Regression equation model 1: muscle strength = 18.21 + 0.945 * ASMI. Regression equation model 2: muscle strength = 24.61 + 1.29 * ASMI—0.24 * body fat percentage.

**significant at P < 0.01

**Table 3:** Results of multiple linear regression modelling on the relationship between muscle strength, mass and body fat percentage in older males.

Model	Coefficient (B)	Standard error B	95% CI	Standardised β	R^2^	P-Value
**Model 1**					0.055**	0.006
**Constant**	24.921	5.834	13.380, 36.461			
**ASMI**	1.806	0.652	0.515, 3.097	0.235		
**Model 2**					0.097**	< 0.001
**Constant**	29.814	6.066	17.813, 41.815			
**ASMI**	2.276	0.669	0.953, 3.599	0.296		
**%BF**	- 0.370	0.152	- 0.670, - 0.071	- 0.213		

ASMI = appendicular skeletal muscle mass index. %BF = body fat percentage. Regression equation model 1: muscle strength = 24.92 + 1.81 * ASMI. Regression equation model 2: muscle strength = 29.81 + 2.28 * ASMI—0.37 * body fat percentage.

**significant at P < 0.01

When exploring the association between muscle strength and muscle mass according to obesity classification using body fat percentage, muscle mass was significantly associated with muscle strength in non-obese males and females. However, in participants with obesity, muscle mass was no longer associated with muscle strength (Tables 4 and 5).

**Table 4:** Results of multiple linear regression modelling on the effect of obesity in the relationship between muscle strength and mass in older females.

Exploratory variable			Coefficient (B)	Standard error B	95% CI	Standardised β	R^2^	P-Value
**Body fat percentage categories**	Non-obese	Constant	12.34	2.43	7.55, 17.13		0.138	< 0.001**
		ASMI	1.91	0.36	1.20, 2.62	0.371		
	Obese	Constant	21.0	4.50	11.97, 30.02		0.005	0.617
		ASMI	0.304	0.60	- 0.91, 1.51	0.068		

*Significant at P < 0.05

**significant at P < 0.01

**Table 5:** Results of multiple linear regression modelling on the effect of obesity in the relationship between muscle strength and mass in older males.

Exploratory variable			Coefficient (B)	Standard error B	95% CI	Standardised β	R^2^	P-Value
**Body fat percentage categories**	Non-obese	Constant	21.71	6.03	9.78, 33.64		0.083	< 0.001**
		ASMI	2.22	0.68	0.88, 3.57	0.289		
	Obese	Constant	26.32	22.03	- 22.18, 74.81		0.024	0.615
		ASMI	1.20	2.32	- 3.91, 6.32	0.154		

*Significant at P < 0.05

**significant at P < 0.01

## 4. Discussion

In this cross-sectional study, we evaluated the relationship between muscle strength, muscle mass, and body fat percentage in older adults living in Auckland, NZ. The findings indicate that muscle strength was associated with muscle mass. The magnitude of this association was greater in males than females, with addition of body fat percentage slightly increasing the ability of the model to predict muscle strength. When exploring the association between muscle strength and muscle mass according to obesity classification using body fat percentage, muscle mass was associated with muscle strength in non-obese participants. However, this association was not observed in older adults who were classified as obese. This indicates that body fat percentage should be considered when measuring associations between muscle mass and muscle strength in older adults.

### 4.1. Prevalence of obesity, low muscle strength and low muscle mass

We found a higher prevalence of participants with obesity using body fat percentage classifications (18.7%) than using BMI classifications (16.3%). This result was as expected, as BMI has been shown to underestimate adiposity in older adults [37]. A recent survey in New Zealand using BMI classifications reported that the prevalence of obesity in older adults between 65–74 years was 34.9% [38]. The lower level of obesity reported in our population may reflect our recruitment inadvertently targeting healthy older adults.

We also identified 3.5% of participants had low muscle strength. The lack of studies reporting the prevalence of low muscle strength and the application of different cut-off values makes it difficult to compare studies. In this cohort, we applied the updated cut off values of low muscle strength defined by the European Working Group on Sarcopenia in Older People (EWGSOP2). A nationally representative sample of Brazilians aged 65 years and older using the same cut-off values as our study observed a higher prevalence of low muscle strength (28.2%) [18]. Other studies which applied the older cut off values defined by the European Working Group on Sarcopenia in Older People (EWGSOP), observed a higher prevalence of low muscle strength of 33.9% among Mexican people 50 years and older [39], 22.5% among Europeans aged 70 years and older [40], 44% in a population of Americans aged 65 years and older [41] and 71% in a community-dwelling older New Zealanders aged 75 years and older [42]. The higher prevalence observed in these groups, is possibly explained by the inclusion of people older than 74 years, and a potentially less healthy population than those participants included in our study.

The prevalence of low ASMI was 6.3% in females and 2.3% in males in our study using cut-offs of <7 kg/m^2^ and <5.5 kg/m^2^ for men and women respectively. Another study in New Zealand adults aged 56–93 years [34] found that 12% of females and 4% of males had low muscle mass using ASMI cut-offs of <7.2 and <5.4 kg/m^2^ for males and females respectively. The higher percentage in the study appears to be explained by the inclusion of adults over the ages of 74 years.

### 4.2. Association between body composition and muscle strength

Our results provide evidence that muscle mass is positively associated with muscle strength in older men and women. This result aligns with the literature [43, 44] and suggests that efforts to maintain muscle mass should have a significant effect on preserving strength in older adults.

When stratified by sex, we observed strong evidence that muscle mass was significantly associated, but not a major contributor to muscle strength in older men and women. Muscle mass accounted for 5% of the variance in muscle strength in men and 4% in women. This finding was similar to another study which found that leg muscle mass accounted for 5% and 4% of the variance in quadriceps muscle strength in men and women, respectively [44].

In a regression model taking into account muscle mass, it was shown that an increase of 1 unit muscle mass will increase the value of muscle strength by 0.945 kg in females and 1.81 kg in males. After taking muscle mass and body fat percentage into account, %BF increased the ability of the model to predict muscle strength. A decrease of 1% body fat and increase of 1 unit muscle mass was shown to increase the value of muscle strength by 1.53 kg in females and 2.65 kg in males. These results highlight not only the importance of increasing muscle mass, but also the importance of decreasing body fat percentage to preserve muscle strength in older adults. The cross-sectional nature of our data impedes any causal inference. Nevertheless, the results from our study provide justification for further prospective research that evaluates the effects of interventions, which are aimed at optimising body composition and muscle strength in older adults.

### 4.3. The role of obesity classification in the relationship between muscle strength and muscle mass

To our knowledge, this is the first study to investigate the role of obesity classification based on body fat percentage in the relationship between muscle strength and muscle mass. Results from multiple linear regression analyses provide evidence supporting the important role of obesity classification according to body fat percentage when investigating the relationship between muscle strength and muscle mass. Our study demonstrated that when obesity was classified using body fat percentage, muscle mass was significantly associated with muscle strength in non-obese older adults. However, an association between muscle strength and muscle mass was not observed in older adults categorised as obese.

The accumulation of intramuscular lipid content (or poor muscle quality), which is seen in people with obesity may explain the influence of obesity in the relationship between muscle strength and muscle mass. Goodpaster et al. reported that higher intramuscular lipid content is associated with lower muscle strength, independent of muscle mass [45]. Also, accumulation of intramuscular lipid content is known to be associated with insulin insensitivity, inflammation and functional deficits in skeletal muscle. It will be important in the future to continue to focus on understanding predictors of muscle strength in older adults with obesity in order to provide appropriate interventions to increase muscle strength.

There were significant strengths to our study. The relatively large sample size permits us to examine whether the relationship between muscle strength and muscle mass was similar in males and females. Also, it is possible that the inclusion of community-dwelling healthy older adults provides the opportunity to identify issues and promote preventative action in early old age. Furthermore, the use of DXA is an accurate measure of body composition. However, in contrast to magnetic resonance imaging (MRI) or computed tomography (CT) DXA cannot detect intramuscular fat from muscle mass nor distinguish the composition of muscle [46, 47].

This cross-sectional study limits the ability to detect causality; hence, only associations were discussed. Other limitations are the population group, which was not representative of the New Zealand population, as this cohort was composed of a convenience volunteer sample of men and women aged 65–74 years living in the community. The classification by body fat percentage for obesity may also be perceived as a limitation given the arbitrary nature of the cut-off points. Finally, we did not assess lower extremity muscle strength, which is a more direct predictor of falls. However, grip strength is associated with lower-body muscle strength [48] and a strong predictor of disability [49].

## 5. Conclusions

Muscle mass and body fat percentage were predictors of muscle strength in this cohort. Muscle mass was associated with muscle strength in non-obese older adults whereas, there was no association between muscle mass and muscle strength in older adults who were classified as obese. This indicates that obesity classification plays an important role in the relationship between muscle strength and muscle mass in older adults. We suggest that this could be mainly attributed to muscle quality, which could be a contributor of muscle strength in older adults who are obese. Further research should focus on identifying predictors of muscle strength in older adults with obesity.

## Supporting information

S1 File (SAV)
